# Prognostic significance of B7-H4 expression in matched primary pancreatic cancer and liver metastases

**DOI:** 10.18632/oncotarget.12665

**Published:** 2016-10-14

**Authors:** Yun Qian, Yiwen Sang, Frederick X.C. Wang, Bo Hong, Qi Wang, Xinhui Zhou, Tianhao Weng, Zhigang Wu, Min Zheng, Hong Zhang, Hangping Yao

**Affiliations:** ^1^ Department of Clinical Laboratory, The Second Affiliated Hospital, Zhejiang University School of Medicine, Hangzhou 310009, China; ^2^ Department of Bioengineering, Erik Jonsson School of Engineering and Computer Science, The University of Texas at Dallas, Texas 75080, USA; ^3^ Department of Pathology, The Second Affiliated Hospital, Zhejiang University School of Medicine, Hangzhou 310009, China; ^4^ Department of Gynecology, The First Affiliated Hospital, Zhejiang University School of Medicine, Hangzhou 310003, China; ^5^ State Key Laboratory for Diagnosis and Treatment of Infectious Diseases, Collaborative Innovation Center for Diagnosis and Treatment of Infectious Diseases, The First Affiliated Hospital, Zhejiang University School of Medicine, Hangzhou 310003, China; ^6^ Department of Gastroenterology, The First Affiliated Hospital, Zhejiang University School of Medicine, Hangzhou 310003, China

**Keywords:** B7-H4, pancreatic cancer, liver metastases, immunohistochemistry, survival analysis

## Abstract

Liver metastasis development in pancreatic cancer patients is common and confers a poor prognosis. Clinical relevance of biomarker analysis in metastatic tissue is necessary. B7-H4 has an inhibitory effect on T cell mediated response and may be involved in tumor development. Although B7-H4 expression has been detected in pancreatic cancer, its expression in liver metastases from pancreatic cancer is still unknown. In this study, overall 43 pancreatic cancer liver metastases (with matched primaries in 15/43 cases) and 57 pancreatic cancer cases without liver metastases or other distant metastases were analyzed for their expression of B7-H4 by immunohistochemistry. Survival curves and log-rank tests were used to test the association of B7-H4 expression with survival. B7-H4 was highly expressed in 28 (65.1%) of the 43 liver metastases and 9 (60.0%) of the 15 matched primary tumors. The expression of B7-H4 in liver metastases was significantly higher than in the matched primary tumors (*p* < 0.05). Patients with high B7-H4 expression in their primary pancreatic cancer had higher risk of developing liver metastases (*p* < 0.05). In univariate analysis, B7-H4 expression was significantly associated with the risk of death (*p* < 0.05). And the multivariate analysis identified that B7-H4 was an independent prognostic indicator (*p* < 0.05). Our results revealed B7-H4 to be associated with poor prognosis in patients with pancreatic cancer liver metastasis. B7-H4 may promote pancreatic cancer metastasis and was promising to be a potential prognostic indicator of pancreatic cancer.

## INTRODUCTION

Pancreatic cancer is one of the most fatal malignancies, while the five-year survival rate of about only 5% [1]. Pancreatic cancer was the seventh leading cause of cancer death worldwide in 2012. According to the National Central Cancer Registry of China (NCCR), the rates of pancreatic cancer incidence and mortality in 2011 were 5.96/100,000 and 5.40/100,000, respectively, ranking tenth and sixth, respectively, among all cancers in China in that year [2].

The prognosis for pancreatic carcinoma is poor. Liver metastasis is often occurred in 70% of patients with pancreatic cancer. Occult liver metastases may already have been present at the time of surgery are one of the most common sites of treatment failure and are associated with poor prognosis [3–7]. In recent years, the median survival time of patients with pancreatic cancer liver metastases is < 6 months [8–11]. Once liver metastases are present, curative resection is not possible. Thus, we should pay more attention on liver metastases to define the mechanisms of them and to identify patients with pancreatic cancer who have great risk of progressing them [12].

B7-H4 (B7S1 or B7X) is a member of B7 family [13–15] and inhibits the T cell mediated response by inhibiting T cell proliferation, activation, and cytokine production [13, 16, 17]. B7-H4 also inhibits the innate immune response by suppressing the growth of immunocyte, thereby allowing tumors to avoid immunologic surveillance [13, 18]. B7-H4 was overexpressed in many tumors, including non–small cell lung cancer, melanoma, breast, ovary, prostate, esophagus, stomach, pancreas, and kidney, but not in normal tissues. B7-H4 may be associated with cancer progression and can be a prognostic marker in some tumors [19, 20].

Although our primary research has found that B7-H4 was expressed in pancreatic carcer, and high expression level of B7-H4 was positive correlation with lymph node metastasis [21, 22], there were no reports on its expression in liver metastases from pancreatic carcinoma. In the present research, we detected B7-H4 expression in matched primary pancreatic and metastatic liver tumors through immunohistochemistry and aimed to determine its clinical significance in liver metastases.

## RESULTS

### Clinical characteristics of pancreatic cancer patients with liver metastases

A total 43 patients (26 men and 17 women) with liver metastases from pancreatic cancer were enrolled in the research. All patients were followed until May 2016 when 43 patients were deceased. All tumor types of the primary pancreatic cancer were infiltrating ductal adenocarcinoma. The median age was 66 years (range: 36–82 years) at the time of transliver biopsy. Table 1 summarizes the patients' characteristics.

**Table 1:** Characteristics of patients with liver metastases

Characteristic	Number (%)
Age (years)	
Median	66
Range	36–82
Gender	
Male	26 (60.5%)
Female	17 (39.5%)
Differentiation	
Well	5 (11.6%)
Moderately	17 (39.5%)
Poorly	21 (48.8%)
Treatment	
Chemical treatment	23 (53.5%)
None	20 (46.5%)

### B7-H4 expression in primaries and liver metastatic pancreatic cancers

B7-H4 expression was high in 28 (65.1%) of 43 liver metastases and nine (60.0%) of 15 matched primary pancreatic cancers. The clinical significance of B7-H4 expression in liver metastases was analysed through comparing the expression level of B7-H4 with patients' clinical characteristics. There was no significant relationship between B7-H4 expression and age, sex, differentiation, or treatment modality (Table 2).

**Table 2:** Correlation between B7-H4 expression and clinical characteristics of patients with liver metastases

Characteristic	Number	B7-H4		*P*-value
		High (%)	Low (%)	
Age (years)				*P* > 0.05
< 65	20	14 (70.0%)	6 (30.0%)	
> = 65	23	14 (60.9%)	9 (29.1%)	
Gender				*P* > 0.05
Male	26	15 (57.7%)	11 (42.3%)	
Female	17	13 (76.5%)	4 (23.5%)	
Differentiation				*P* > 0.05
Well	5	2 (40.0%)	3 (60.0%)	
Moderately	17	11 (64.7%)	6 (35.3%)	
Poorly	21	15 (71.4%)	6 (28.6%)	
Treatment				*P* > 0.05
Chemical treatment	23	14 (60.9%)	9 (39.1%)	
None	20	14 (70.0%)	6 (30.0%)	

### Differential expression of B7-H4 in primaries and liver metastatic pancreatic cancers

**Figure 1:**
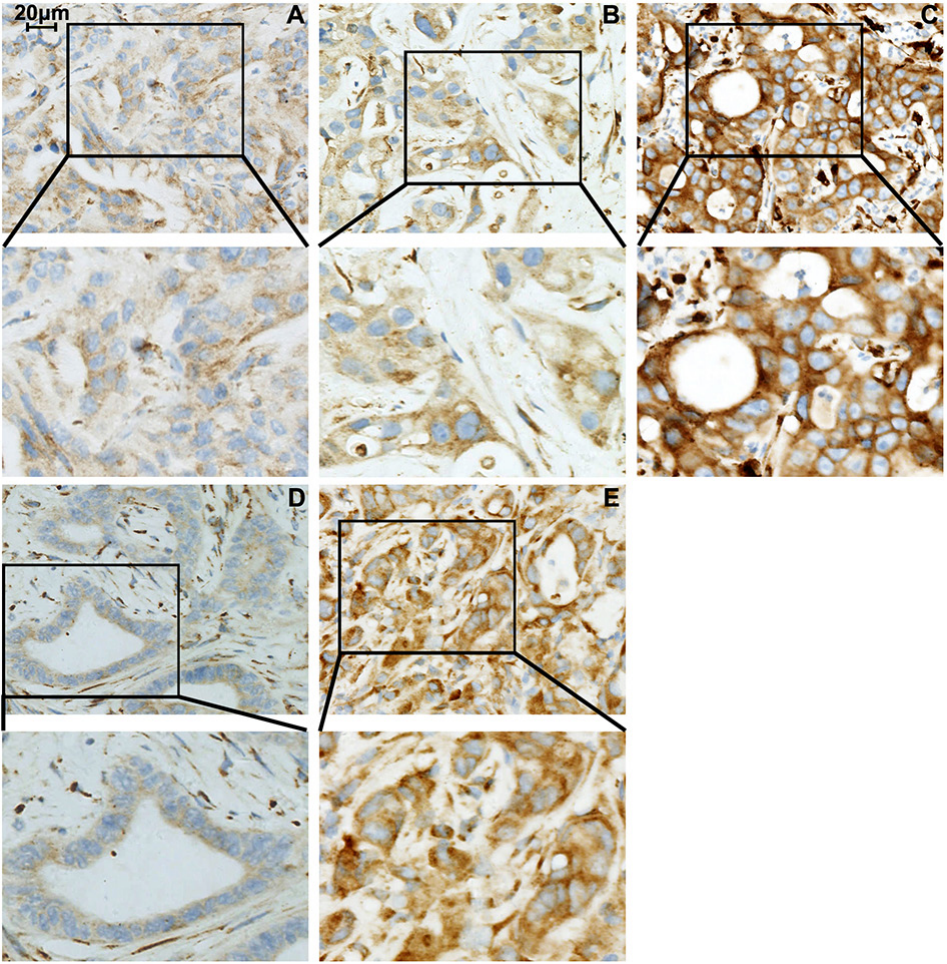
B7-H4 expression in liver metastases from pancreatic cancer (**A**) weak staining (staining intensity score = 1); (**B**) moderate staining (staining intensity score = 2); (**C**) strong staining (staining intensity score = 3). Enhanced B7-H4 expression in liver metastases (**E**) as compared with the matched primary pancreatic cancer (**D**) Note: Magnification for all photomicrographs is ×400.

**Figure 2:**
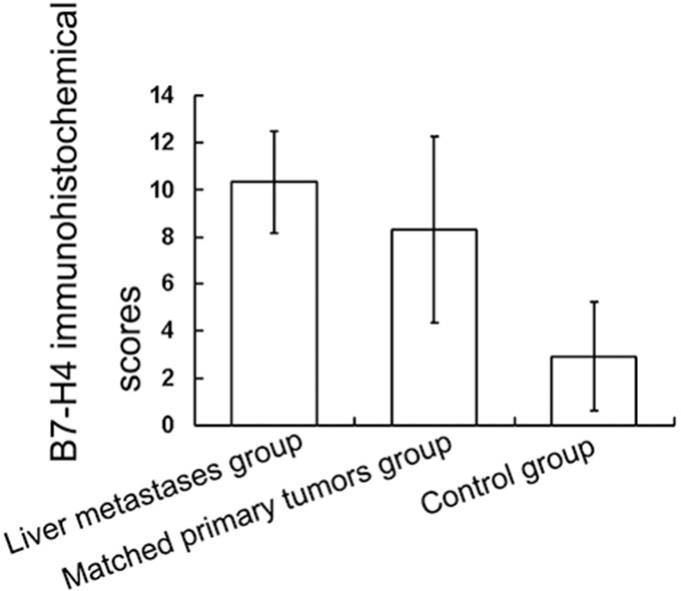
Differential expression of B7-H4 in liver metastases and primary pancreatic tumors The immunohistochemical score of B7-H4 expression in the liver metastases was higher than in the matched primary pancreatic cancer in 15 paired cases *(p* < 0.05*)*. And the immunohistochemical score of B7-H4 expression in the matched primary pancreatic cancer was higher than in the control group *(p* < 0.05*)*.

We measured the differential expression of B7-H4 through immunohistochemical stain score criterion between 15 paired tumors (Figure 1A–1C). In 10 cases, the expression of B7-H4 status in liver metastases was consistent with matched primary pancreatic tumors: in 8 cases, the expression of B7-H4 was high, and 2 cases showed low B7-H4 expression. However, in 5 cases, the expression of B7-H4 was higher in liver metastases than in matched primary pancreatic cancers (Figure 1D and 1E). In those 15 paired cases, the expression of B7-H4 in liver metastases was significantly higher than in matched primary tumors (10.33 ± 2.16 vs. 8.30 ± 2.93, *p* < 0.05) (Figure 2).

The differential expression of B7-H4 was also detected by immunohistochemical stain score criterion between the primary pancreatic cancers and control. The matched primary pancreatic cancer scores were significantly higher than that of the pancreatic cancer without liver metastases or other distant metastases diagnosed during the same period (8.30 ± 2.93 vs. 3.94 ± 2.29, *p* < 0.05; Figure 2).

### Prognostic value of B7-H4 in liver metastases from pancreatic cancer

**Figure 3:**
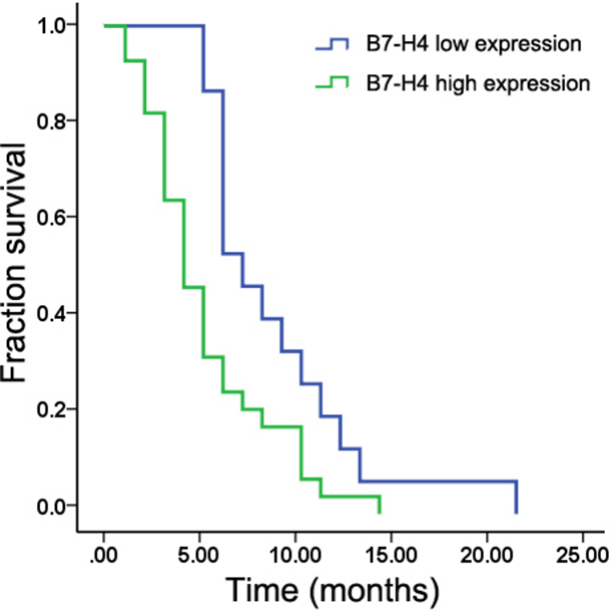
Kaplan–Meier analysis showing that patients with liver metastases with high B7-H4 expression had shorter survival than those with low expression (*p* < 0.05)

We determined the role B7-H4 played in survival using Kaplan–Meier analysis and log rank test. The median overall survival (OS) was 6.0 ± 0.5 months (95% confidence interval [CI]: 5.1–6.9 months). The median OS for patients who had high expression of B7-H4 in liver metastases was 4.0 ± 0.6 months (95% CI: 2.9–5.1 months) as compared with a median OS of 7.0 ± 0.8 months (95% CI: 5.4–8.6 months) for patients who had low expression of B7-H4 (*p* < 0.05) (Figure 3). Tables 3 and 4 list the univariate survival analysis results, where low B7-H4 expression and chemotherapy treatment were positively correlated with survival (*p* < 0.05) and were independent prognostic factors in the multivariate analysis.

**Table 3:** Univariate analysis of OS

Characteristic	Cases	Median Survival (months)	95% (CI) (months)	*P*-value
Age (years)				*P* = 0.745
< 65	20	5.0 ± 0.7	3.5–6.5	
> = 65	23	6.0 ± 0.6	4.9–7.1	
Gender				*P* = 0.436
Male	26	6.0 ± 0.5	5.0–7.0	
Female	17	5.0 ± 0.8	3.4–6.6	
Differentiation				*P* = 0.517
Well	5	8.0 ± 3.3	1.6–14.4	
Moderately	17	6.0 ± 0.7	4.7–7.3	
Poorly	21	5.0 ± 0.8	3.5–6.5	
Treatment				*P* = 0.023
Chemical treatment	23	6.0 ± 1.0	4.1–7.9	
None	20	4.0 ± 0.6	2.7–5.3	
B7-H4 expression				*P* = 0.012
High	28	4.0 ± 0.6	2.9–5.1	
Low	15	7.0 ± 0.8	5.4–8.6	

**Abbreviations:** CI, confidence interval.

**Table 4:** Multivariate analysis of OS

Variable	RR	95% CI	*P*-value
Age	0.790	0.344–1.812	0.578
Gender	0.622	0.272–1.421	0.260
Differentiation	0.587	0.179–1.928	0.380
Treatment	0.405	0.193–0.851	0.017
B7-H4 expression	0.489	0.240–0.997	0.049

**Abbreviations:** CI, confidence interval; RR, relative risk.

## DISCUSSION

Recently, advances in biotechnology and immunologic techniques had led to progress in research for anti-tumor immunological therapy. B7-H4 is a significant costimulatory molecule responsible for T cell inactivation [20], which is widely expressed in many tumor tissues and possibly acts as a negative regulatory factor in anti-tumor immune response [23, 24]. The aberrant expression of B7-H4 in tumor tissues may be used as a tumor marker or therapy target for immunotherapy [20, 25, 26]. Our previous findings show evidence of B7-H4 expression in pancreatic cancer tissues [21, 22]. Given the high incidence of liver metastases, we attempted to elucidate the effect of B7-H4 in liver metastases from pancreatic cancer here.

In this research, we found that the expression of B7-H4 was higher in liver metastases compared with matched primary pancreatic cancers. In addition, we measured the expression of B7-H4 in a patient series with pancreatic cancer with or without (control) liver metastases, demonstrating that the matched primary pancreatic cancer with liver metastases had higher B7-H4 expression than control. The relative effect of B7-H4 on survival in patients with liver metastases was also determined. The aberrant expression of B7-H4 in liver metastases indicated poorer survival. Thus, B7-H4 may be a prognostic factor for pancreatic cancer patients with liver metastases.

B7-H4 inhibits both innate immunity and T cell responses in the tumor microenvironment [27]. B7-H4 overexpression had greater prognostic significance and promotes tumor tolerance, and it might inhibit the function of antigen-presenting cells and promote regulatory T cells (Tregs) proliferation and development [26]. The aberrant expression of B7-H4 in tumor cells was also negatively correlated with CD8+ T cell density in the tumor stroma. B7-H4 knockdown increased CD8+ T cell–mediated cytotoxicity *in vitro*, suggesting that B7-H4 expression may shield tumors from immune surveillance by suppressing the tumor-infiltrating CD8+ T lymphocytes in the tumor microenvironment [23, 28]. B7-H4 overexpression in tumor was related to the increased immune escape. However, the precise role of B7-H4 in T cell regulation and the underlying mechanisms remain to be fully elucidated. Further researches are required to explore the specific role of B7-H4 in pancreatic cancer.

In addition, B7-H4 might be a target for immunotherapy in tumor [29]. Therapeutics targeting B7-H4 could produce significant synergistic outcomes, eliminating cancer cells and favorably altering the tumor microenvironment, as B7-H4 was expressed on tumor cells and tumor-associated macrophages in various cancers. It was reported that T cell function and augmented T cell mediated responses was successfully rescued *in vivo*, and the tumor burden reduced in a murine tumor model using anti-mouse B7-H4 antibodies, indicating proof of therapy for targeting B7-H4 *in vitro* [13, 15, 30]. Although the researchers had provided insight into the mechanism of B7-H4 promoting tumor escape from immune surveillance in the last decade, there was no targeting B7-H4 therapies investigated clinically [31]. There remain many opportunities for determining combinatorial therapies for targeting or overcoming B7-H4-mediated T cell hypofunction in the tumor microenvironment can bring clinical benefit to patients with relative cancer risk.

The differential expression of B7-H4 in primary tumors and metastases indicated that B7-H4 was positively associated with tumor development and poor survival [32, 33]. B7-H4 suppressed tumor-specific T cell mediated responses and promoted the infiltration of immunosuppressive T cells such as Tregs into the lungs in an experimental murine model of lung metastasis. Consequently, B7-H4 knockout mice had less lung metastases than wild-type B7-H4 mice [34]. High B7-H4 expression level was positively associated with local invasion, poorer differentiation, distant metastasis, and lymph node metastasis [35], and was independent prognostic factors of OS [36]. Our study discovered discordance in expression of B7-H4 between the liver metastases and the matched primary pancreatic cancer. Higher expression level of B7-H4 in liver metastases might indicate a suppressed effect on anti-tumor immune responses, the development of liver metastases, and warrant more close surveillance. Our results indicated that B7-H4 might be a potential diagnostic and prognostic marker and immunotherapy target for pancreatic cancer.

In conclusion, our results found that the expression level of B7-H4 was higher in liver metastases than in matched primary pancreatic cancer. The high B7-H4 expression in liver metastases was positively correlated to poorer prognosis. Additionally, B7-H4 might promote progression and metastasis of pancreatic cancer and was promising to be a potential prognostic indicator of pancreatic cancer.

## MATERIALS AND METHODS

### Patients and tissue specimens

We analyzed 43 patients pathologically diagnosed with liver metastases from pancreatic cancer between January 2000 and December 2015 at The Second Affiliated Hospital, Zhejiang University School of Medicine. All patients underwent transliver biopsy for liver metastases resection. In addition, 15 matched corresponding primary pancreatic cancers were obtained. The clinical parameters included patient demography, tumor differentiation, and treatment modality. Fifty-seven patients with pancreatic cancer who were treated during the same period without liver metastases or other distant metastases were enrolled as the control group. All tissues were fixed in 10% buffered formalin and embedded in paraffin. Two pathologists reviewed all archival hematoxylin and eosin–stained sections. The Ethics Committee of The Second Affiliated Hospital, Zhejiang University School of Medicine approved the present study.

### Immunohistochemical staining

Immunohistochemical staining was performed using the two-step EnVision method (Dako, Glostrup, Denmark). Paraffin-embedded tissues were cut into 5-μm serial sections, transferred onto adhesive slides, and dried at 65°C for 2 hours. The sections were deparaffinized with xylene and rehydrated through graded alcohols. Endogenous peroxidase activity was blocked with 0.3% hydrogen peroxide solution for 30 minutes at room temperature and antigen retrieval was performed at 100°C for 30 minutes in citrate buffer (10 mmol/L; pH 6.0). After washing three times with PBS at 5 minutes each time, the sections were incubated with 10% normal goat serum to block nonspecific binding. Then, the sections were incubated with rabbit anti-human B7-H4 monoclonal antibody (1:400; clone number EP1165; Abcam, Cambridge, MA, USA) at 4°C overnight, followed by immunodetection using the Dako EnVision detection system (K5007). The slides were counterstained with Mayer's hematoxylin, dehydrated in graded alcohol, and mounted with a neutral resin. The negative control was performed by replacing the primary antibody with PBS. Human tonsil tissue was used as the positive control.

### Evaluation of B7-H4 staining

Two pathologists without knowledge of the patients' clinical records examined and scored the sections. Five tumor fields at ×400 magnification were randomly selected. Cytoplasmic and/or tumor cell membrane staining were considered to indicate positive expression. A previously reported semiquantitative system [16] was used to determine B7-H4 expression. The proportion of positive cells was scored as follows: 0 (< 5%); 1 (6%–25%); 2 (26%–50%); 3 (51%–75%); 4 (> 75%). Staining intensity was evaluated as follows: 0 (no staining); 1 (weak staining, light yellow); 2 (moderate staining, yellowish brown); 3 (strong staining, brown). The sum score, determined by multiplying the positive proportion score by the intensity score, was as follows: 0 (negative); 1–4 (weakly positive); 5–8 (moderately positive); 9–12 (strongly positive). In this study, B7-H4 expression was considered low and high when the score was < 9 and ≥ 9, respectively.

### Statistical analysis

Statistical analysis was performed using SPSS (v17.0; IBM Corporation, Armonk, NY, USA). The relationship between B7-H4 expression and clinicopathological characteristics was compared using the chi-square test or two-tailed Fisher's exact test. McNemar's test for paired data was used to compare the frequency of B7-H4 expression between liver metastases and the matched primary tumors. The two independent samples *t*-test was used to analyze the significance of B7-H4 expression scores between the matched corresponding primary pancreatic cancer group and the control group. The OS was calculated from the diagnosis of liver metastases until death or the date of the last follow-up. Survival data were analyzed by the Kaplan–Meier method and log rank test. Cox proportional hazard model analysis was used to identify the independent prognostic factors of survival. *P* < 0.05 was considered statistically significant.
